# Physical characterization, biocompatibility, and antimicrobial activity of polyvinyl alcohol/sodium alginate blend doped with TiO_2_ nanoparticles for wound dressing applications

**DOI:** 10.1038/s41598-024-55818-8

**Published:** 2024-03-05

**Authors:** Manar A. Ibrahim, G. M. Nasr, R. M. Ahmed, Nermeen A. Kelany

**Affiliations:** 1 Physics Department, Faculty of Science, Zagazig University, Zagazig, 44519 Egypt; 2 Physics Department, Faculty of Science, Cairo University, Giza, Egypt

**Keywords:** Wound dressing, Titanium dioxide, Polyvinyl alcohol, Sodium alginate, Biophysics, Biomaterials

## Abstract

The ability of wound dressing materials to tackle skin pathogens colonization that is associated with open wound infections is limited. Recently, green-synthesized metal oxide nanoparticles has received a lot of attention to overcome this limitation. However, titanium dioxide nanoparticles (TiO_2_-NPs) exhibit exceptional antibacterial properties. In this work, several concentrations (0, 1, 3, and 5 wt.%) of TiO_2_ NPs prepared using *Aloe vera* leaf extract were added to a blend of polyvinyl alcohol and sodium alginate (PVA:SA). This nanocomposite was designed to enhance the healing process of wounds. The interaction between the PVA:SA composite and the TiO_2_ NPs was confirmed by FTIR. The thermal behavior of the nanocomposite films was investigated using DSC and TGA. The experimental results indicate that the glass transition temperatures of the nanocomposites increased by increasing the added amount of TiO_2_ NPs to be 53.7 °C (1 wt.%), 55.8 °C (3 wt.%), and 60.6 °C (5 wt.%), which were consistently lower than the glass transition temperature of the matrix material (69.6 °C). The Dynamic Mechanical Analysis was examined. The nanocomposite doped with 5 wt.% of TiO_2_ NPs detected a high storage modulus (21.6 × 10^8^). Based on swelling and degradation studies, the prepared PVA:SA:TiO_2_ nanocomposite films have an excellent swelling rate, and the inclusion of TiO_2_ NPs increases the stability of the polymeric matrix. The PVA:SA:TiO_2_ nanocomposite films exhibited a superior antibacterial efficacy against Gram-positive bacteria such as *Bacillus cereus* and *Staphylococcus aureus*, compared to their effectiveness against Gram-negative bacteria like *Escherichia coli*. Moreover, the nanocomposite films were biocompatible with Human Skin Fibroblast. Therefore, the developed PVA:SA:TiO_2_ nanocomposite films suit wound dressing applications.

## Introduction

Wound has become an integral aspect of our being. This may result in skin injury, such as lacerations, ruptures, or tissue inflammation. Microorganisms such as bacteria can cause infection in open wounds. Furthermore, this will also proliferate to adjacent healthy tissues^1^. Wound healing occurs in an organized sequence of overlapping phases that results in tissue reconstitution. The formation of mature scar tissue indicates the end of this process, which also includes hemostasis, inflammation, and proliferation^2^.

Wound dressing is an ancient and popular method to motivate wound healing. The conventional dressings, such as gauze and cotton, have the benefits of being highly absorbent and inexpensive. However, they only contribute passively to the healing process by simply isolating the wound from the contaminations^3^. Nevertheless, conventional dressings maintain the wound area dry and promote adhesion to the wound, which can cause discomfort and pain for the patient as well as delaying the healing of the wound^4^. Furthermore, they are unable to protect the wound from microbial invasion, which cause the desiccation of wound, the wound exudates build up on the surface of wound, and insufficient gas permeability^5^. On the other hand, some pathogens that cause skin infections have become resistant to the most widely used antibiotic creams. To overcome such limitations, the idea of creating a humid environment surrounding the wound zone and finding a promising antibacterial nanoparticles to treat pathogen-caused skin diseases are essential for the manufacturing and design of modern wound dressings^6^.

Numerous biopolymers have recently been studied to develop wound dressing materials^7^. Alginate is one of these polymers that have attracted much interest because of being cheap, biocompatible, biodegradable, and well-tolerated by the immune system^8^. Moreover, because of its high hydrophilicity, alginate may effectively absorb excess exudate and provide a moist environment during wound healing^9^. Alginate is an anionic polysaccharide derived from brown algae. It has a linear structure comprising two 1–4 linked residues of β D mannuronate and α L guluronate^10^.

Alginate film is rarely used alone, despite its ability to film-forming, because of its low mechanical strength and thermal stability^11^. A composite material addresses this issue by combining sodium alginate with a pliable polyvinyl alcohol (PVA), forming a patch^12^. Incorporating natural polymers into PVA is a subject of study due to its potential to enhance desired physical characteristics and biocompatibility. Polyvinyl alcohol (PVA) is a biocompatible polymer that exhibits semi-crystalline properties. It possesses desirable features such as excellent flexibility, high transparency, significant hydrophilicity, and solubility in hot water.

Additionally, PVA demonstrates notable chemical stability^13^. Due to its inherent biocompatibility with bodily organs, polymeric material finds extensive utilization in various biomedical applications, including but not limited to artificial intestines, artificial blood arteries, contact lenses, and drug-delivery systems^14^. PVA has also been found to be an effective material for wound dressings^15^.

An optimal wound dressing material should possess several key characteristics. Firstly, it should create an optimal moist environment around the wound. Additionally, it should effectively absorb any excessive exudates emanating from the wound. Moreover, it should exhibit antimicrobial properties to prevent infection. Furthermore, the material should be biocompatible, ensuring compatibility with the body's tissues. It is also crucial that the material should be noncytotoxic namely, it should not be harmful to healthy cells^16^. Thus, a recent approach involving incorporating a biocompatible filler into the wound dressing material was proposed to address this issue. In order to enhance the mechanical characteristics of biopolymers, nanobiocomposites are created by including inorganic metal oxide nanoparticles as fillers. These nanoparticles do not only improve the qualities of the material, but also confer bioactivity to an otherwise inert substance^17^. Furthermore, nanofillers improve physico-chemical properties of nanobiocomposites because of their high surface area and interaction with polymer chains at the molecular level^18^.

The antimicrobial properties were determined to titanium oxide^19^. Other researchers pointed out that TiO_2_ can be an efficient filler material in biodegradable polymers because it promotes cell attachment and proliferation^20^. It has medical and health applications because of its inert properties. In contrast, titanium dioxide (TiO_2_) is often considered a highly favorable choice for an effective inorganic material due to its exceptional biocompatibility and biomechanical properties. At present, various medicines are available in the market and are frequently used to assist in the healing process of wounds. Glucocorticoid steroids and non-steroidal anti-inflammatory and chemotherapeutic drugs are examples of such medicines^21^. The available therapeutic medicines for treating wounds are costly, hazardous, and harmful to surrounding healthy tissues. This stimulated research on green-synthesized nanoparticles with different morphologies as a bactericidal agent to overcome open wound ulcers. Several studies have investigated the use of different types of green synthesized metal oxide NPs, such as TiO_2_, ZnO, CuO, and ZnO/CuO, in various applications^22–26^.

The green synthesis technique is advantageous compared to chemical procedures because it avoids dangerous reducing agents. Instead of relying on expensive chemical reagents, plant extracts and microorganisms are utilized, resulting in less toxicity and improved suitability for biomedical applications^27^. Interestingly, some green synthesized metal-based NPs exhibit antioxidant properties and promote wound healing. It is believed that antioxidants accelerate wound healing by reducing oxidative stress in the wound^28^. It was generally expected that low-temperature annealed green-synthesized nanoparticles would have excellent medicinal properties and show constant, low reactive oxygen species (ROS) release that is safe and nontoxic to cell lines^29^.

An inquiry into utilizing green TiO_2_ production revealed noteworthy wound-healing activity in an albino rat. This was substantiated through the implementation of wound closure measurements, histological analysis, and protein expression profiling^30^. According to Chen et al.'s findings, using a titanium dioxide (TiO_2_) membrane in polyurethane has demonstrated remarkable characteristics, rendering it very suitable as a material for wound dressings^31^. In addition, for use in wound healing, Archana et al.^32^ created a film and bilayer composite using nanostructured TiO_2_. Results of their research demonstrated that the incorporation of CS (chitosan) and pectin into a nanostructured TiO_2_ (titanium dioxide) matrix resulted in enhanced tensile strength, favorable physiochemical characteristics, favorable biocompatibility, potent antibacterial activity, and expedited wound healing. Ismail et al.^33^ conducted a study to evaluate the potential of using a biofilm composed of gellan gum (GG) combined with titanium dioxide nanoparticles (TiO_2_-NPs) as a wound dressing material. The researchers discovered that the GG + TiO_2_ NPs biofilm has strong antibacterial properties against *Escherichia coli* and *Staphylococcus aureus* germs. Furthermore, the biofilm formed by combining GG and TiO_2_ NPs exhibited enhanced cell-to-cell contact capabilities, leading to increased cell migration and proliferation. This, in turn, facilitated the faster healing of open excision wounds in Sprague Dawley rats. It was investigated that the potential use of polymer blends based on SA/PVA loaded with nanoparticles in wound dressing applications^16^. The authors of the present study adhered to the prevailing research trajectory.

The present study involved preparing a microenvironment conducive to wound healing by combining SA and PVA encapsulated with different concentrations (0, 1, 3, and 5 wt.%) of greenly synthesized TiO_2_ NPs using *Aloe vera* leaf extract. The current study encompassed the examination of FTIR, thermal analysis (TGA and DSC), and the -DMA properties of the nanocomposite films that were created. The nanocomposites were characterized by investigations of their degradation studies and swelling properties. The antimicrobial activity of the prepared nanocomposite films was also evaluated against the predominant pathogens of both Gram-positive and Gram-negative bacteria, as well as yeast and fungi. The produced nanocomposites were assessed for cytotoxicity using the Sulforhodamine-B (SRB) test.

## Experimental techniques

### ***Preparation of PVA:SA:TiO***_***2***_*** nanocomposite films***

Firstly, TiO_2_ NPs were synthesized in a previous work^34^ by green route using extract of *Aloe vera* and TiCL_4_ as a precursor. Briefly, 100 ml of extract from *Aloe vera* leaves was gradually added to a 100 ml 1.0 N TiCL_4_ solution in deionized water under constant stirring. The mixture's pH was adjusted at 7 and stirring was maintained at room temperature for 4 h. After being filtered and washed using double-distilled water, the formed nanoparticles were left to dry overnight at 100 °C. Finally, the obtained dry powder was calcined at 500 °C for 4 h.

Secondly, in a previous study conducted by the authors, PVA:SA:TiO_2_ nanocomposite films were created utilizing a solvent-casting process^35^. In summary, a homogeneous solution was obtained by mixing and constantly stirring PVA and SA solutions (PVA: SA 3:1 w/w) for a duration of 6 h. After a short time of sonication, several suspensions of TiO_2_ nanoparticles (NPs) with concentrations of 0 wt.%, 1 wt.%, 3 wt.%, and 5 wt.% by weight were added to the pre-prepared solution of PVA and SA. The suspensions were agitated for an extended period, after that transferred into Petri dishes, and left to evaporate.

### Fourier transform infrared (FT-IR) spectroscopy

The chemical structure of the materials was determined using the Fourier Transform Infrared Spectroscopy (FT-IR) technique, specifically employing the Shimadzu Prestige-21 Spectrophotometer. The FT-IR spectra were obtained in the wavenumber range from 4000 cm^−1^ to 600 cm^−1^.

### Differential scanning calorimeter (DSC)

The thermal transition behavior was investigated using the differential scanning calorimeter model Shimadzu DSC-50 for all the nanocomposites. The measurement was conducted in a range from room temperature to 230 °C. The experiment used a heating rate of 10 °C/min in a nitrogen environment.

### Thermal gravimetric analysis (TGA)

Mettler Toledo TA-TGA was used to measure thermal stability by investigating the thermal gravimetric analysis (TGA). The samples were heated from room temperature to 593 °C, under N_2_ atmosphere, with a heating rate of 10 °C/min.

### Dynamic mechanical analysis (DMA)

Nanocomposites were analyzed for their thermo-mechanical properties using dynamic mechanical analysis (DMA). Triton Instruments were used to conduct the tests on the nanocomposites at frequencies of 0.5, 1, 3, and 5 Hz. The readings were analyzed in tension mode at a heating rate of 10 °C/min, from room temperature to 150 °C, by measuring the temperature dependence properties such as the loss and storage moduli and the loss factor (tan δ). The prepared samples were of 0.14 mm thickness.

### Zeta potential analysis

The Zeta potential analysis of the nanocomposite films composed of PVA:SA:TiO_2_ was conducted using dynamic light scattering (DLS) techniques with a Zeta sizer instrument manufactured by Malvern (obtained from the United Kingdom). The specimens were immersed in distilled water and subjected to sonication in a bath sonicator to achieve a uniform dispersion. Zeta potential measurements were conducted under ambient conditions at 25 °C and a detection angle of 90 degrees.

### Swelling and degradation studies

Pieces of a uniform weight were cut from the prepared nanocomposite films. Each sample was placed into a sterilized plastic container containing a phosphate buffer saline (PBS) solution of pH 7.4 and incubated at 37 °C for different time intervals up to 14 days. The samples were removed from the incubating solution, surface wiped, and weighed to determine their wet weights (W_w_) at the predetermined time (1, 3, 5, 7, 9 and 14 days). They were then dried in an oven at a temperature of 40 °C for forty-eight hours until they reached a steady weight. Subsequently, the specimens were reweighed in order to ascertain their dry weights (W_d_).

The swelling percentage was calculated using the following formula:

1 $${\text{DS}}\% = \frac{{{\text{W}}_{{\text{w}}} - {\text{W}}_{{\text{d}}} }}{{{\text{W}}_{{\text{d}}} }} \times 100$$

DS denotes the swelling degree, whereas W_w_ and W_d_ provide the weights of the wet and dry film, respectively^36^. On the other hand, the weight loss percentage of the sample was recorded using the following equation^37^:

2 $${\text{Weight}}\;{\text{loss}}\% = \frac{{{\text{W}}_{{\text{i}}} - {\text{W}}_{{\text{d}}} }}{{{\text{W}}_{{\text{i}}} }} \times 100$$

where W_i_ is the initial weight of each sample.

### Biological activity of the nanocomposites

#### Antimicrobial activity using agar diffusion technique

The antibacterial activity of the materials was evaluated against five indicator microorganisms using the agar diffusion method^38^. Standard and clinically isolated microorganism strains were used such as *Aspergillus Niger*, Bacillus cereus, *Staphylococcus aureus*, *Candida albicans*, and *Escherichia coli*. The tested organisms (10^6^ colony-forming units/ml) were inoculated overnight in potato dextrose agar of yeast and fungi and nutrient agar media of bacteria, then poured promptly into sterile Petri dishes. Subsequently, samples were cut into small parts (1 × 1 cm^2^) and placed onto the agar plate surface. After 24-h incubation at the optimum growth temperatures for the inoculated plates, the diameter of the inhibition zone was measured in centimeters. In the present study, Streptomycin was used as standard antibacterial (positive control), while Fluconazole acted as standard anti-fungal. 10% (v/v) of dimethyl sulfoxide (DMSO) acted as a negative control.

#### Determination of antimicrobial activity (MIC) using broth media

The antimicrobial activity of the four different membrane concentrations was assessed in vitro. The growth inhibition of pathogenic organisms was measured using standard and clinically isolated microorganism strains, such as *Bacillus cereus* (Gram-positive bacteria), *Staphylococcus aureus* (Gram-positive bacteria), *Candida albicans* (yeast), and *Aspergillus Niger* (fungi) and *E. coli* (Gram-negative bacteria)^39^. The samples were cut into small pieces (0.03 g) and put into tubes containing 10 mL of potato dextrose broth (fungi) or nutrient broth (bacteria and yeast) inoculated with 100 µl of each microorganism. Both the vaccinated and conventional tubes (without samples) were kept at their optimal growth temperatures for a period of twenty-four hours. The temperature for bacteria was 37 °C, while the temperature for yeast and fungi was 28 °C. Streptomycin was used as standard antibacterial (positive control), while Fluconazole served as standard anti-fungal. 10% (v/v) of (DMSO) act as a negative control. A spectrophotometer was utilized in order to determine the optical densities (O.D.) of the microbial growth at a wavelength of 625 nm. Experiments were carried out in duplicate for every strain of microorganisms that were put down for examination. These findings were reported in the form of an average value.

3 $$Inhibition\;of\;microbial\;growth(\% ) = 100 - \left[ {\frac{O.D\;of\;sample(trail)}{{O.D\;of\;sample(s\tan dard)}} \times 100} \right]$$

### Cytotoxicity assay

The Sulforhodamine-B (SRB) assay was used to evaluate the in vitro cytotoxicity of the prepared samples against HSF: Human Skin Fibroblast. Nawah Scientific Inc., (Mokatam, Cairo, Egypt) provided the HSF. An amount of 10% (v/v) of (DMSO) was adapted as strong cytotoxic material (negative control), while, human skin fibroblast cells without addition of PVA:SA:TiO_2_ nanocomposite films (untreated cells) were used as blank control in the experiment. Aliquots of 100 μL cell suspension (5 × 10^3^ cells) were in 96-well plates and incubated for 24 h in complete media. Cells were treated with another aliquot of 100 μL media containing the prepared samples at various concentrations (50 and 100 μg/ml). After 72 h of samples' exposure, cells were fixed by adding 150 μL of 10% trichloroacetic (TCA) to the medium and incubating for one hour at 4 °C. After removing the TCA solution, the cells were washed five times with distilled water. Aliquots of 70 μL SRB solution (0.4% w/v) were added and incubated for 10 min in a dark place at room temperature^40^. The plates were air-dried overnight after being washed 3 times with 1% acetic acid. To dissolve the protein-bound SRB stain in the plates, 150 μL of 10 mM tris(hydroxymethyl) aminomethane (TRIS) was added^41^. A BMG LABTECH- FLUO star Omega Microplate Reader (Ortenberg, Germany) was used to measure the absorbance at 540 nm. Equation (4) was utilized to calculate the cell viability of the treated cells.

4 $$\begin{aligned} & {\text{Cell}}\;{\text{viability }}\left( \% \right)\;{\text{of}}\;{\text{ treated}}\;{\text{ cells}} \\ & \quad = [{\text{Absorbance}}\;{\text{measured}}\;{\text{for}}\;{\text{treated}}\;{\text{cells}}/{\text{Absorbance}}\;{\text{measured}}\;{\text{for}}\;{\text{the}}\;{\text{control}}\;{\text{untreated}}\;{\text{cells}}] \, \times \, 100 \\ \end{aligned}$$

### Statistical Analysis

The GraphPad Prism's two-way ANOVA was used to determine statistical significance. If the P value was less than 0.05, the results were considered statistically significant (p < 0.05). Data are presented as mean values ± standard deviation (SD).

## Results and discussion

### FTIR analysis

**Figure 1:**
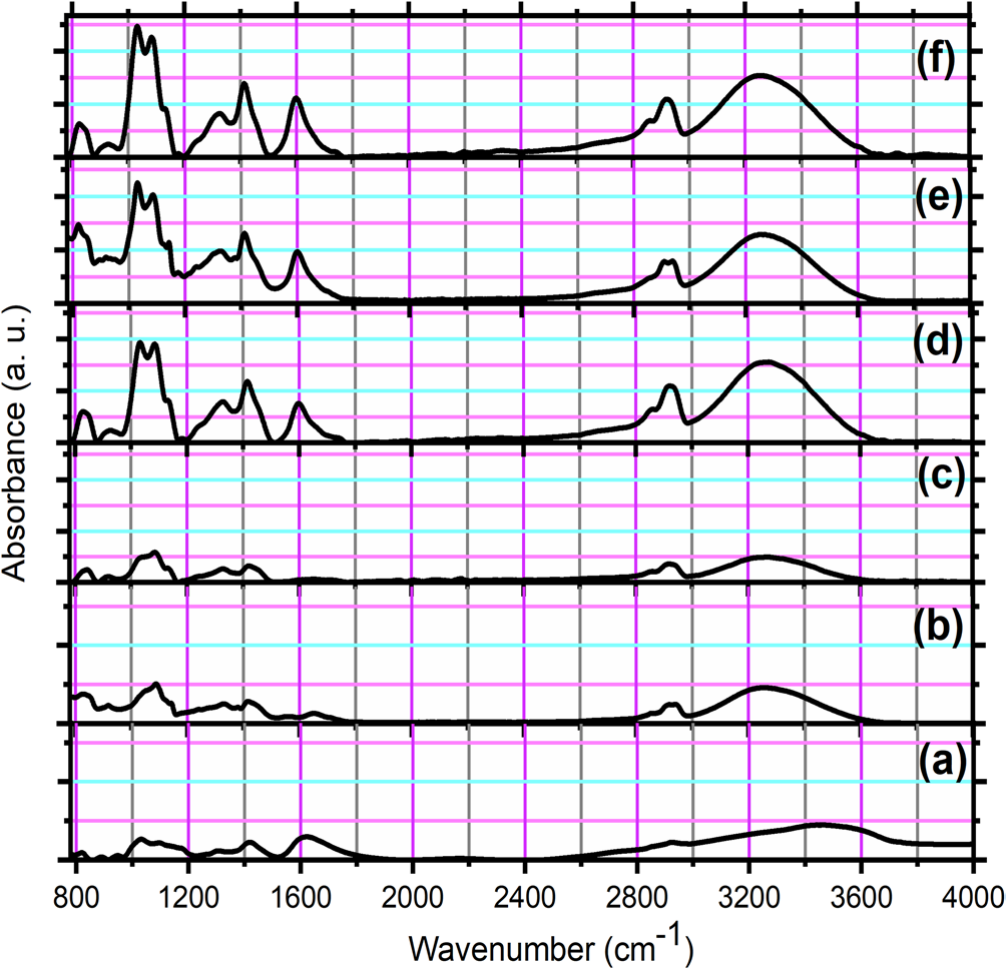
FTIR spectra of (**a**) SA, (**b**) PVA, (**c**) PVA:SA blend, and the nanocomposites with different concentrations of TiO_2_ NPs doped in the blend (**d**) 1 wt.%, (**e**) 3 wt.%, and (**f**) 5 wt.%.

With the information it gives on the blend's composition and the interactions between the polymers of the studied blend (PVA and SA) and the doped fillers (TiO_2_ NP_S_), FTIR deserves a place among the essential methods for the characterization of systems. Figure 1 illustrates the FT-IR spectra of SA, PVA, the blend of PVA: SA 3:1, and the nanocomposites with different concentrations of TiO_2_ NPs doped in the blend (1, 3, and 5 wt.%). The band at 834 cm^−1^ was assigned to unsaturated CH_2_ stretching of PVA (Fig. 1b) which suffered a slight shift in its position after blending it with SA (Fig. 1c) to be at 840 cm^−1^, and after adding TiO_2_ NPs (Fig. 1d–f) with 1 wt.% (831 cm^−1^), 3 wt.% (820 cm^−1^) and 5 wt.% (826 cm^−1^). The IR band at 1028 cm^−1^ and 1088 cm^−1^ are assigned to C–O–C stretching vibration of SA^42^ (Fig. 1a), and C–O stretching bond of PVA^43^ (Fig. 1b), respectively. These two bands suffered from a slight change in their positions to be (a shoulder at 1031 and 1083) cm^−1^, (1032 and 1087) cm^−1^, (1030 and 1084) cm^−1^, and (1024 and 1084) cm^−1^ corresponding to PVA: SA blend, and 1, 3 and 5 wt.% of the TiO_2_ NPs doped in the blend, respectively, (Fig. 1d–f). The positions of CH_2_ asymmetric stretching of SA at 2927 cm^−1^ (Fig. 1a), and the wavenumber of 1409 cm^−1^ were assigned to the stretching vibrations of Ti–O-Ti of TiO_2_NPs (Fig. 1d–f), the bending C-H of PVA (Fig. 1a). The symmetric stretching vibrations of –COO^–^ of SA (Fig. 1b)^44^ were changed, after being blended and doped with varying TiO_2_ NP concentrations of 1, 3, and 5wt.% of TiO_2_ NPs, to be at 2920, 2922, 2908 and 2920 cm^−1^, respectively, and at 1420 cm^−1^, 1414 cm^−1^, 1412 cm^−1^ and 1413 cm^−1^, respectively. In addition, the asymmetric –COO^–^ stretching vibrations of SA at 1645 cm^−1^ (Fig. 1a) also related to the bending band of adsorbed water molecules on the surface of the nanoparticles, changed to be at 1645 cm^−1^, 1597 cm^−1^, 1599 cm^−1^ and 1598 cm^−1^, corresponding to PVA:SA blend, and 1, 3 and 5 wt.% of the TiO_2_ NPs doped in the blend, respectively, which is in agreement with the literature^45^. The bands observed at 3260 ± 4 cm^−1^ in all the studied samples were assigned to the stretching vibration of -OH groups of SA^46^. The IR band at 1325.5 ± 1.5 cm^−1^ was ascribed to the stretching vibration of Ti–O–Ti of TiO_2_ NPs^47^ and also to C–O asymmetrical stretching of SA^48^. Changes in the fingerprint regions of the IR spectrum, as well as in the shape and intensity of bands at a range of 1597–1645 cm^−1^, at 1028 ± 4 cm^−1^, at 1085 ± 2 cm^−1^, at 1325.5 ± 1.5 cm^−1^, and at a range of 1412–1420 cm^−1^, were observable. These results can be consequences of TiO_2_ NPs and the potential interaction of PVA and SA at varying concentrations of NPs^49^.

### DSC analysis

**Figure 2:**
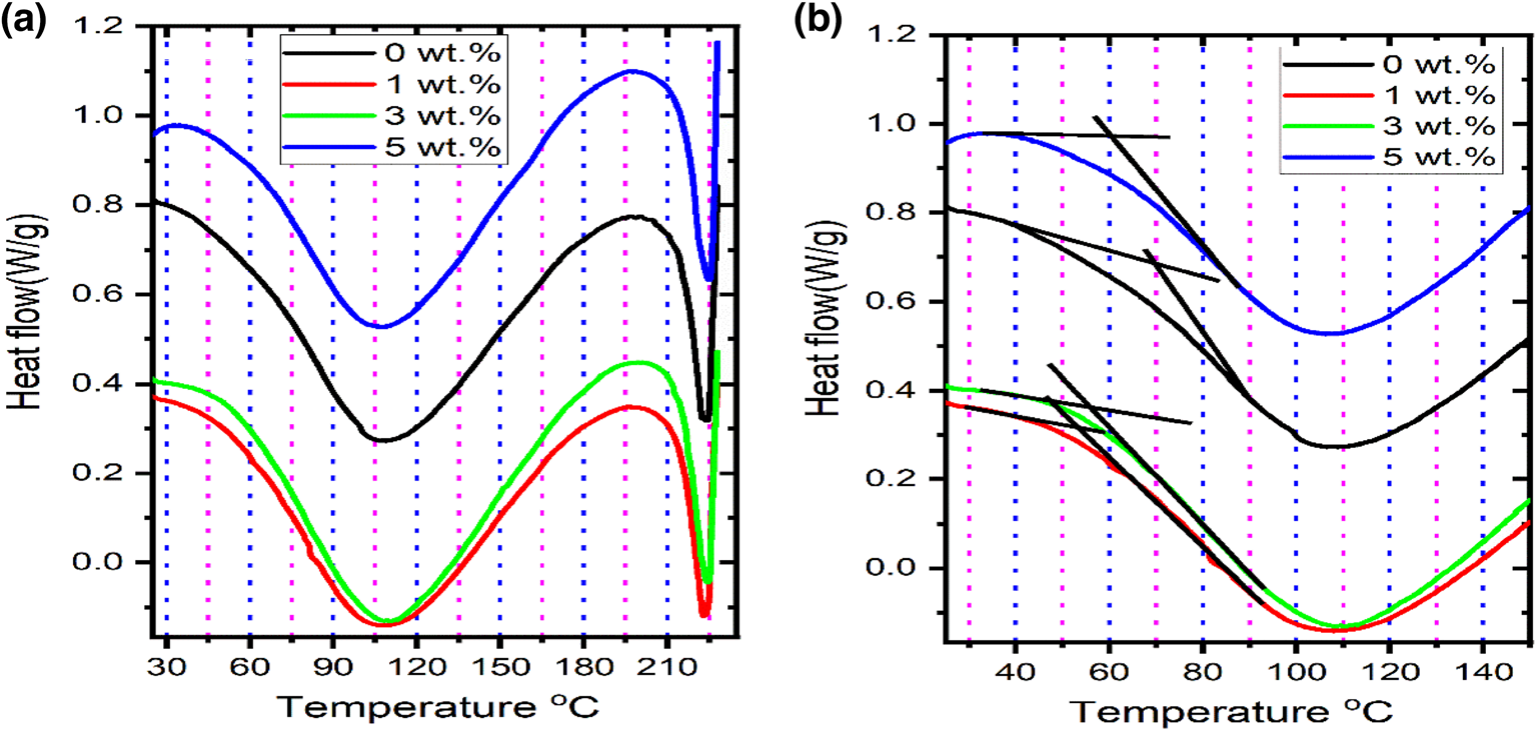
DSC thermograms of different concentrations of TiO_2_ NPs doped in the blend of PVA:SA 3:1(**a**)from 30 °C to 230 °C, and (**b**) a magnification of DSC (from 30 °C to 150 °C).

DSC measurements can yield helpful information regarding materials' thermal behavior and phase transitions. Glass transition and melting are two significant polymer phenomena influenced by the processing environment and any additives added to the hosting matrix. Figure 2a illustrates the DSC thermograms, in a range of temperature from room temperature to 230 °C, for the nanocomposites of PVA:SA:TiO_2_ NPs. For all the nanocomposites, the broad endothermic peaks observed at 108 ± 1 °C were attributed to the evaporation of the nanocomposites' water content^50^. The melting temperature of the PVA: SA 3:1 blend was detected at 223.2 °C, this is in agreement with the literature^51^.

Moreover, this endothermic peak can be attributed to the biopolymer's degradation (Sodium alginate) and the production of the corresponding carbonate^52^. By augmenting the quantity of TiO_2_ NPs incorporated into the host matrix, a marginal shift in the melting temperature was seen, resulting in a value of 225.4 °C when the concentration of TiO_2_ NPs reaches 5 wt.%. This change in the melting temperature can point to an expansion of the hosting matrix's free volume^53^. In addition, a minor reinforcement was accomplished by adding TiO_2_ nanoparticles to the PVA:SA 3:1 blend. Enthalpy (∆H), one of the most significant fundamental properties of materials, is temperature-dependent and changes in its value owing to phase shift are likewise temperature-dependent. By integrating across the region of the DSC curve where the phase transition occurs, the enthalpy change (∆H) for that phase transition may be calculated^54^. The values of the integrated melting temperature peak give melting enthalpy, ∆H_m_ of 30.04 J/g, 27.91 J/g, 27.69 J/g, and 29.80 J/g, corresponding to 0, 1, 3, and 5 wt.% of TiO_2_ NPs doped in the hosting matrix, respectively. Therefore, adding TiO_2_ NPs to the PVA/SA blend reduced its enthalpy.

The glass transition temperature (T_g_) of all the nanocomposites was detected in Fig. 2b. The detected T_g_ value of pure PVA: SA 3:1 blend is 69.6 °C, which is close to the glass transition temperature of the blend of PVA: SA 80:20 (72 °C) published in the literature^55^. The values of Tg of the nanocomposites increased by increasing the added amount of TiO_2_ NPs to be 53.7 °C (1 wt.%), 55.8 °C (3 wt.%), and 60.6 °C (5 wt.%). In addition, all the glass transition temperatures of the nanocomposites containing different concentrations of TiO_2_ NPs were less than that corresponding to the hosting matrix (PVA:SA 3:1 (w/w)).

### Thermogravimetric Analysis (TGA)

**Figure 3:**
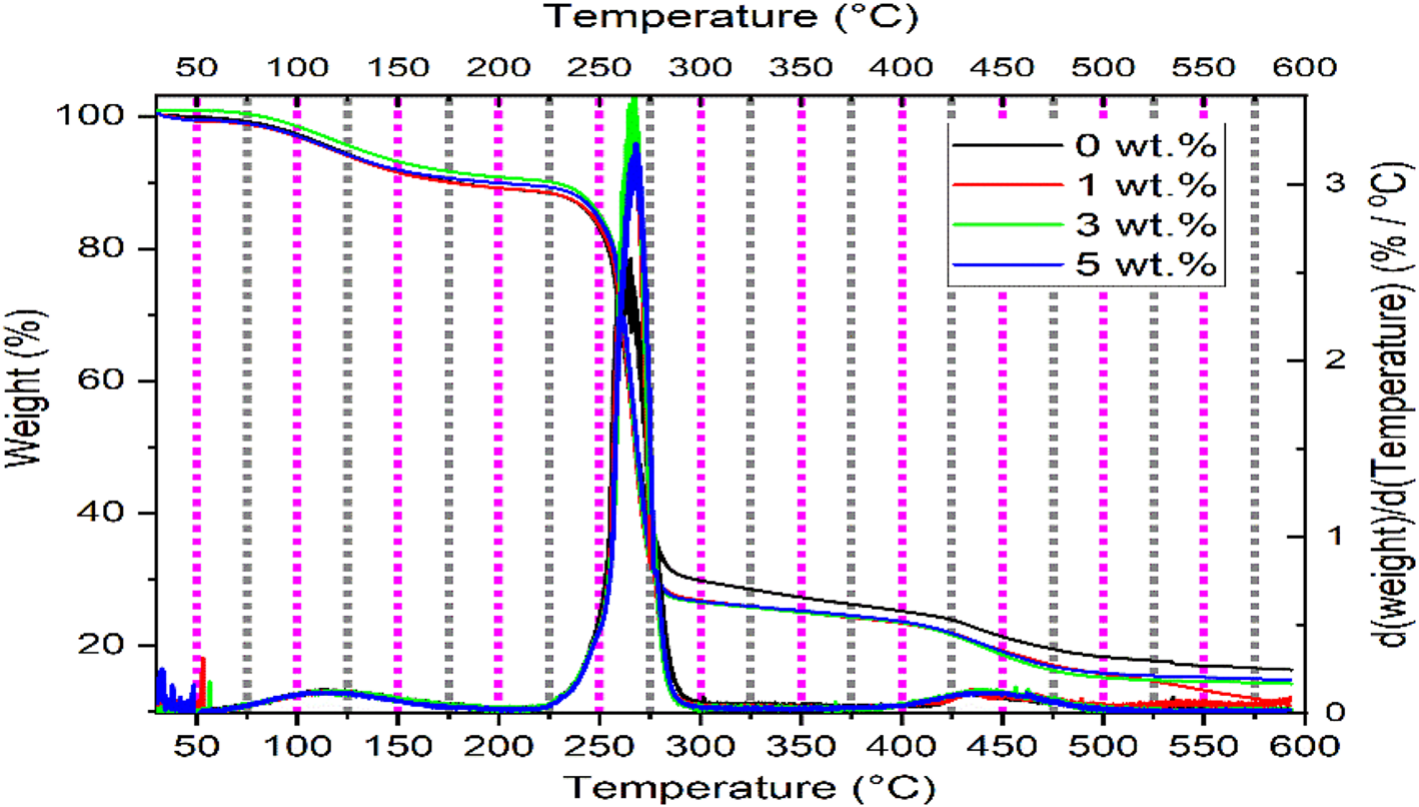
TGA and DrTGA of different concentrations of TiO_2_ NPs doped in the blend of PVA: SA 3:1.

Figure 3 and Table 1 display the thermal degradation behavior of all nanocomposites, of which thermogravimetric analysis (TGA) was examined. Three distinct stages detected [1st stage from 69 °C to176 °C], [2nd stage from 225 to 283 °C], and [3rd from 426 °C to176 °C], can describe the mass loss of the different nanocomposites of PVA: SA: TiO_2_.

**Table 1:** Thermal degradation profiles of the nanocomposites of PVA:SA:TiO_2_ NPs.

The concentration of TiO_2_ NPs doped in PVA: SA (wt.%)	Mass loss (%) at 176 °C 1st Stage [69–176 °C ]	Mass loss (%) at 283 °C 2nd Stage [225–283 °C ]	Mass loss (%) at 498.9 °C 3rd Stage [405 ± 5 °C–176 °C ]	Residual mass (%) at 593 °C
0	9.8	68.0	80.6	16.3
1	10.1	71.7	83.3	10.5
3	8.3	72.4	84.4	14.2
5	9.3	72.1	83.4	14.9

The evaporation of the absorbed water can explain the first stage, in which about 8.3–10.4% of the masses of the nanocomposites were lost. The first stage of decomposition was followed by thermal stability up to the beginning of the second stage at ≈ 225 °C. At 283 °C, the second stage of decomposition, between 68% and 72.4% of the mass was lost. It is known that sodium alginate belongs to the polysaccharide polymers, which are composed of one carboxylate segment and two hydroxyl segments per repeating unit^53^. Therefore, the decomposition of the second stage is ascribed to a complex polysaccharide ring destruction mechanism. Thermal stability occurs after the second stage up to 400 °C for nanocomposites with TiO_2_ NPs and 420 °C for the hosting blend, where the third stage starts. Approximately 80.8%– 84.4% of the mass loss was observed at 498.9 °C during the third stage of decomposition. The mass loss observed in the third stage may be due to the decomposition and dehydration of the SA and PVA backbones^56^.

In addition to a sharp peak temperature corresponding to the second stage of decomposition, Fig. 3 illustrates the DrTGA curves, which exhibit two broad peaks corresponding to the first and third decomposition regions. Furthermore, all the nanocomposites have a thermal stability region after the third stage except for the nanocomposite doped with 1 wt.% of TiO_2_ NPs, which keeps deteriorating. About 10.2–16.3% residue was left at 593 °C, for all the studied samples due to the conversion of sodium alginate into sodium carbonate^56^, as seen in Table 1. Table 1 illustrates that the recorded values of the residual mass (%) for 1, 3, and 5 wt.% of TiO_2_ NPs doped in the PVA:SA blend were 10.5, 14.2, 14.9, respectively. Consequently, the addition of TiO_2_ NPs to the blend of PVA:SA 3:1 (wt.%) caused an increase in the thermal stability of the studied nanocomposites as a result of the strong interaction between the TiO_2_ NPs and the blend^57^. This finding is in a good agreement with some literatures^58,59^.

### Dynamic mechanical analysis (DMA)

#### Storage modulus and loss modulus

**Figure 4:**
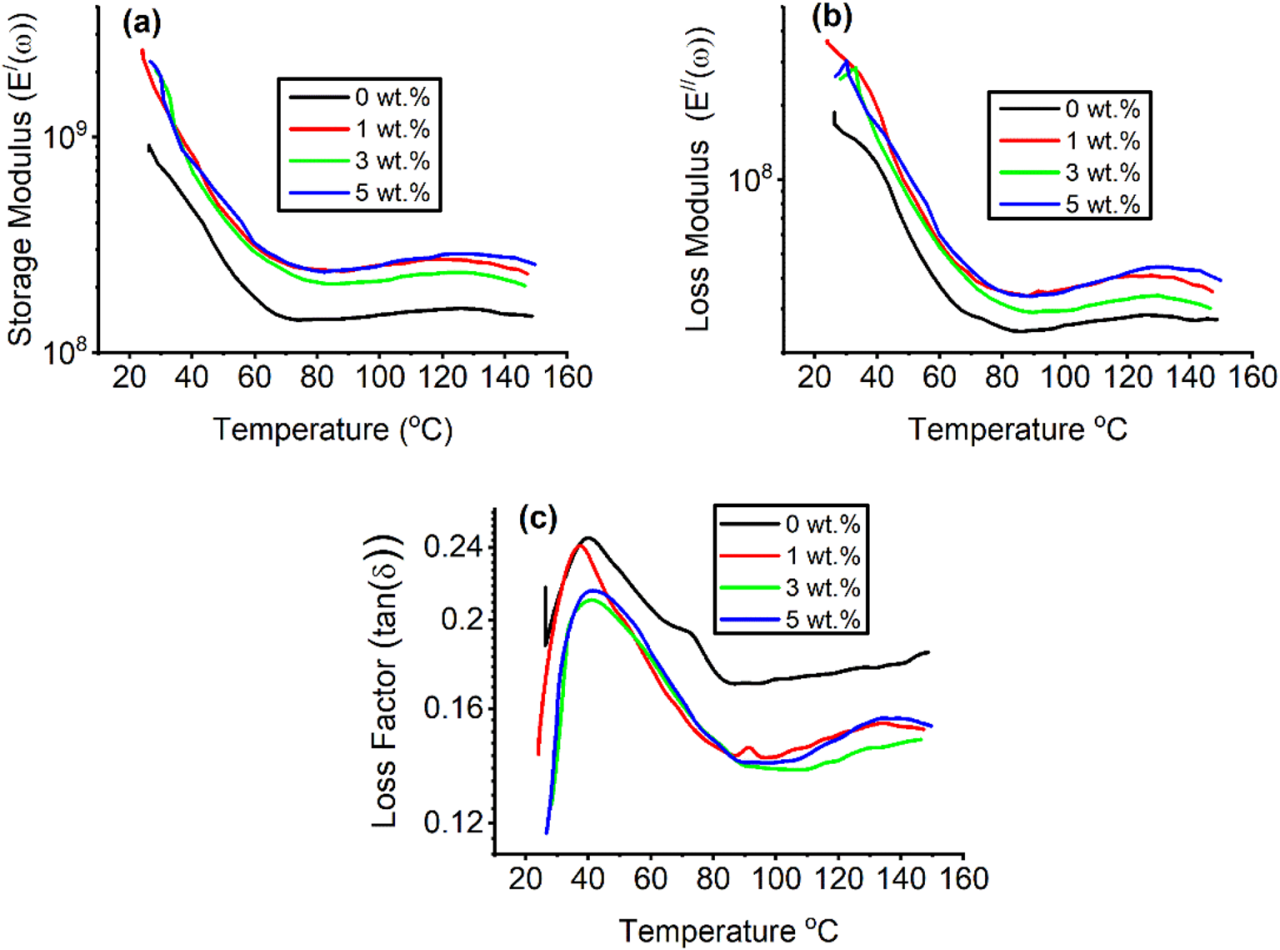
Variation of (**a**) the storage modulus (E^/^(ω)), (**b**) the loss modulus (E^//^(ω)), and (**c**)loss factor (tan (δ)) with the temperature at a constant frequency of 5 Hz.

At 5 Hz, Fig. 4a presents the storage modulus (E^/^ (ω)) of the different concentrations of green synthesized TiO_2_ NPs (0, 1, 3, and 5 wt.%) doped in PVA:SA 3:1 (w/w). The storage modulus decreased with increasing temperature owing to expanding the chain mobility of the polymer blend^60^. At a constant temperature of 28 and 5 Hz, the storage modulus has values of 7.9 × 10^8^, 16.6 × 10^8^,20.4 × 10^8^, and 21.6 × 10^8^, corresponding to 0, 1, 3, and 5 wt.% of TiO_2_ NPs, respectively, doped in PVA:SA blend, (Fig. 4a). The increasing behavior of the storage modulus with TiO_2_ NPs doping indicated that TiO_2_ NPs (wt.%) decrease the space of crosslinking net point^61^. The nanocomposite doped with 5 wt.% of TiO_2_ NPs detected a high storage modulus. The movements of the polymer chains were restricted as they were inserted between the TiO_2_ NPs. Figure 4b shows the loss modulus (E^//^ (ω)) of all nanocomposites at a constant frequency of 5 Hz. The loss modulus followed the same trend as that of the storage modulus. The nanocomposite doped with 5 wt.% of TiO_2_ NPs showed a higher loss modulus than the others. Due to the increase in the free movement of the polymer blend chains with temperature, the loss modulus curves decrease monotonically. The energy was dissipated by friction between TiO_2_ and the polymer blend matrix due to their interactions^62^. These interactions were also evident from the FTIR findings.

#### Loss factor tan (δ)

The ratio of energy dissipated to energy stored is tangent of the phase angle δ, which is given by:

5 $$\tan \delta = \frac{{E^{\prime \prime } (\omega )}}{{E^{\prime } (\omega )}}$$

From the DMA measurements of the complex modulus mode, the glass transition temperature (T_g_) can be determined as temperature is increased at a constant heating rate. Therefore, the potential of a material to dissipate and absorb energy is determined by the loss factor. The variation in the tanδ values, measured for all the nanocomposites over a range of temperatures is shown in Fig. 4c, as an example for the other samples. The neat PVA:SA blend had the highest loss factor value at the glass transition region, as shown in Fig. 4c. However, as the TiO_2_ NPs doping increased, the height of the loss curve decreased. The increased stiffness of the doped nanocomposites could limit the degree of freedom of the blend chain at an atomic level^63^. Moreover, a tan (δ) peak shift to higher temperature with TiO_2_ NPs content was also found in Fig. 4c. The higher the percentage of TiO_2_ NPs, the higher the tan (δ) peak shifts are.

Both glass transition temperature and crosslinking density were associated with the tan δ peak. A wider peak as detected for nanocomposites doped with 5 wt.% of TiO_2_ NPs indicated more time for relaxation of molecules due to decreasing blend chain mobility resulting from the formation of crosslinking density in the blend matrix^64^. The T_g_ for the PVA: SA blend is 39.9 °C, which is in agreement with that reported by Yang et al.^65^, and shifted to a higher temperature for higher TiO_2_ NPs contents, as shown in Table 2.

**Table 2:** The glass transition temperature (T_g_) at different applied frequencies and the activation energy (∆E) for the nanocomposites of PVA:SA:TiO_2_ NPs.

The concentration of TiO_2_ NPs doped in PVA: SA (wt.%)	T_g_ (°C)				∆E (J. mol^−1^)	R^2^
	0.5 Hz	1 Hz	3 Hz	5 Hz		
0	35.9	37.2	39.5	39.7	448.8	97.5
1	34.3	35.3	37.3	37.4	532.0	96.9
3	38.2	38.8	40.7	41.4	588.5	96.2
5	37.0	38.4	39.4	40.7	501.3	96.3

#### Frequency dependence

Several researchers^66^ were interested in studying the effect of frequency on the dynamic mechanical response of polymers. Arrhenius equation was used to elucidate the effect of temperature on the frequency of the glass transition relaxation. This effect of temperature on frequency is described in the following form:

6 $$\upsilon = \upsilon_{o} \exp \left( {\frac{ - \Delta E}{{RT}}} \right)$$

where υ_o_ is a pre-exponential factor, ∆E is the activation energy and R is the universal gas constant^67^.

The activation energy of the glass transition temperature, ∆E, can be estimated from the slope of a plot of ln(υ) versus 1000/T_g_ using Eq. (7):

7 $$\Delta E = - R\frac{d\;lnv}{{d\left( {\frac{1000}{{T_{g} }}} \right)}}$$

**Figure 5:**
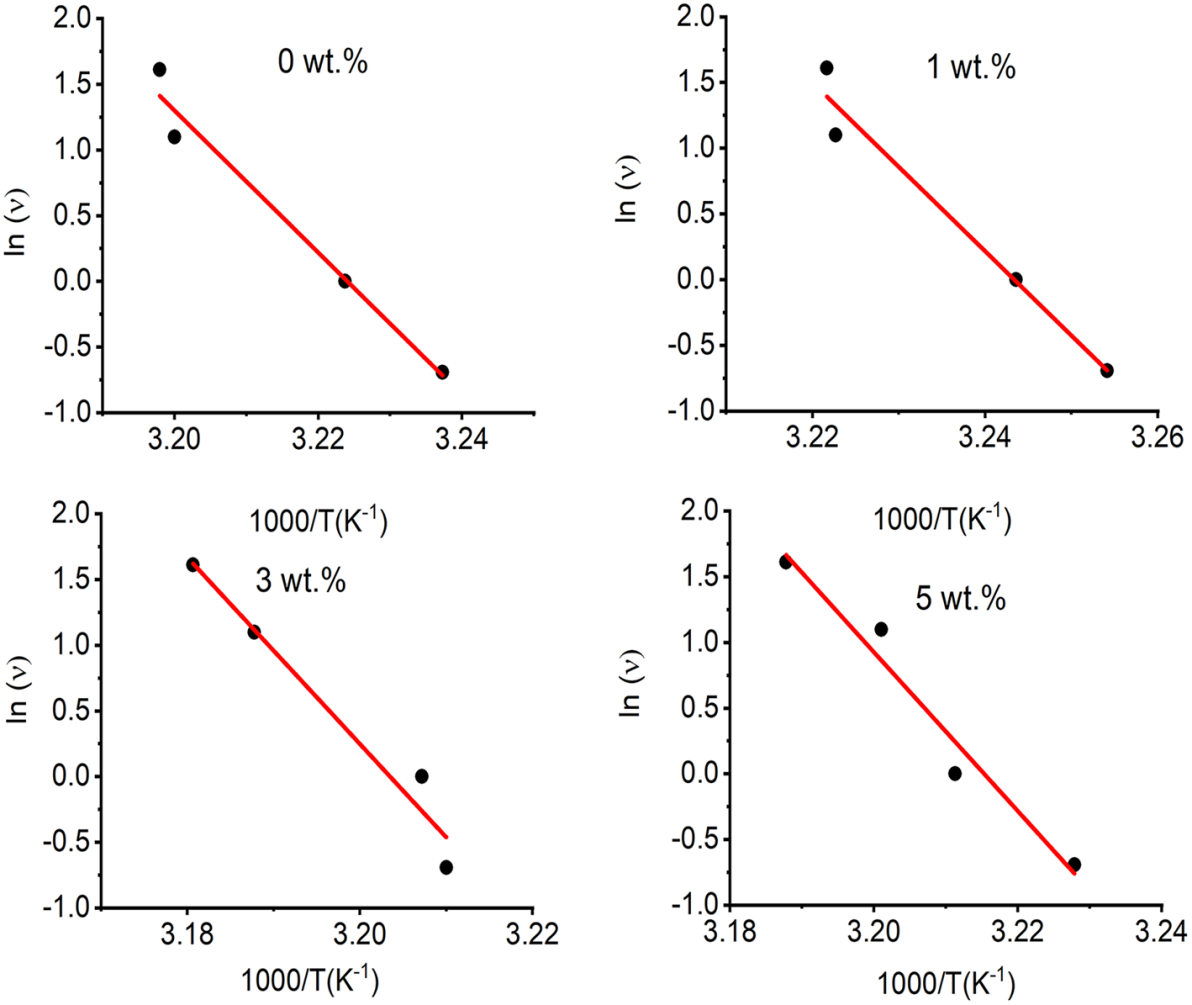
Variation of ln(υ) versus (1000/T) for the nanocomposites of PVA:SA:TiO_2_ NPs.

Figure 5 presents a plot of ln(υ) versus 1000/T_g_, for a heating rate of 10 °C/ min, where T_g_ was determined at the tan (δ) peak position. Table 2 presents the estimated values of the activation energies for all nanocomposites, along with R^2^ values of the regressions for each curve fit. The activation energy with TiO_2_ NPs contents owing to the TiO_2_ NPs imposed stiffness on the composite by reducing the chain mobility.

The relative increase in the modulus slope in the elastomeric region seen in Fig. 4a is attributed to further crosslinking of the polymer matrix at elevated temperatures. The most significant variation in the measured T_g_ is due to frequency changes from 0.5 Hz to 5 Hz was 3.9 °C for the PVA: SA blend. However, the most considerable variation in measured T_g_ of 5wt.% of TiO_2_ NPs was 3.7 °C from 0.5 Hz to 5 Hz. Moreover, the activation energy increases with TiO_2_ NPs doping, indicating the influence of the crosslinking by adding TiO_2_ NPs.

### Zeta potential

The zeta potential (ZP) was measured for TiO_2_ NPs, PVA:SA blend, and the nanocomposites of PVA:SA:TiO_2_ with various contents of TiO_2_ NPs (1, 3, and 5 wt.%) to estimate the colloidal stability of the nanoparticles. The zeta potential of TiO_2_ NPs was highly negative (− 34.1 ± 5 mV), indicating high stability in aqueous solutions and lack of aggregations^68^.

The measured ZP for the PVA:SA blend was highly damaging (− 35.7 ± 9.26 mV), as predictable, because SA and PVA are well known for their anionic nature. It was noted that the PVA:SA blend's zeta potential value was lower than that for the suspension containing only TiO_2_ NPs. However, the zeta potential of the various concentrations of TiO_2_ NPs doped in PVA:SA blend (1, 3, and 5 wt.%) were − 22.2 ± 6.75 mV, − 15.9 ± 4.37 mV, and − 22.5 ± 5.48 mv, respectively. The successful doping of TiO_2_ NPs in PVA:SA blend (1, 3, and 5 wt.%) was convincingly supported by the increased zeta potential values and colloidal stability of the nanoparticles. The increase in zeta potential was attributed to the attractive forces between PVA:SA blend and TiO_2_ NPs by intermolecular interactions. The significant change in zeta potential value may result from consuming TiO_2_ NPs active sites through their chemical reaction with PVA/SA in the prepared PVA:SA:TiO_2_ nanocomposite^69^.

The obtained results from zeta potential for the nanocomposite of PVA:SA:TiO_2_ NPs with 3 wt.% (- 15.9 ± 4.37 mV) was evidence to confirm the successful blending of PVA and SA and doping the formed blend with TiO_2_ NPs which is sufficient to repel other particles, prevent aggregation, and ensures its long-term stability.

### Swelling and degradation studies

#### Swelling behavior of the nanocomposites

**Figure 6:**
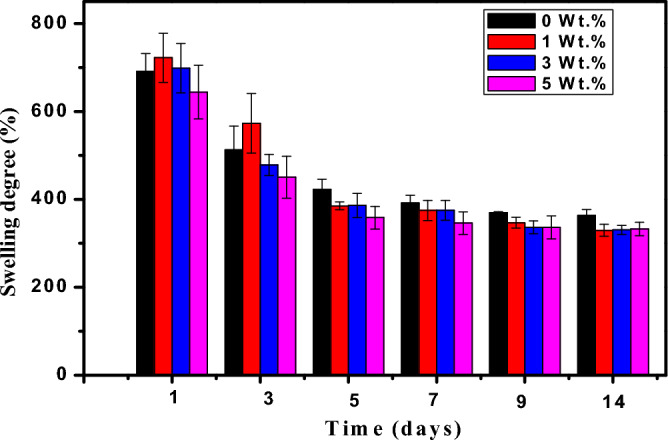
The dependence of the Mean values of swelling percentages for PVA: SA loaded with (0, 1, 3, and 5 wt.% TiO_2_-NPs) on the incubation time.

An essential characteristic of wound dressings is their ability to absorb water^70^. A Swelling study was conducted to assess the prepared nanocomposites' stability and water uptake capability in aqueous PBS media. The nanocomposites showed appreciable uptake of PBS up to 720% at 24 h (Fig. 6). Following this, the water absorption capability decreased and then reached equilibrium after 9 days of immersion in PBS. This decline was probably caused by the collapse of the polymer network following a high water absorption^71^. It was clear from Fig. 6 that the DS% of PVA:SA blend film increases with incorporating TiO_2_ NPs (1wt.%) in the blend. On the other hand, the water absorption ratio decreased with the increase in TiO_2_ NPs content.

The higher swelling properties of PVA:SA:TiO_2_ nanocomposite films compared with PVA:SA blend film may be caused by the hydrophilic nature of TiO2 NPs, PVA, and SA^72^. The reduction of the swelling degree of the nanocomposite films with increasing the content of TiO_2_ NPs (5 wt. %) can be attributed to the probability of creation of Ti–O hydrogen bonds with –OH functional groups, which strengthens the composite structure and limits the mobility of the polymer and inorganic particles, in agreement with the literature^73^. This hindered the penetration of liquid through the polymer matrix. This finding is in a good agreement with the FTIR results. In addition, the following results of the weight loss can confirm these findings.

Based on the (SEM) images of the same investigated samples used in a prior study conducted by the authors^35^, TiO_2_ nanoparticles suffered from agglomeration, which occurred with the increase in its concentration in the host matrix. This might reduce the hydrophilic property of TiO_2_ NPs, which is in agreement with a previous study^74^. Consequently, the amount of the absorbed liquid is reduced. It is necessary to mention that variations in TiO_2_ NPs concentration significantly influenced the swelling behavior of the prepared nanocomposite. The prepared PVA:SA:TiO_2_ nanocomposite films have an excellent swelling rate and can absorb the wound exudates, indicating that the films are appropriate for wound dressings.

#### Weight loss behavior of the nanocomposite

**Figure 7:**
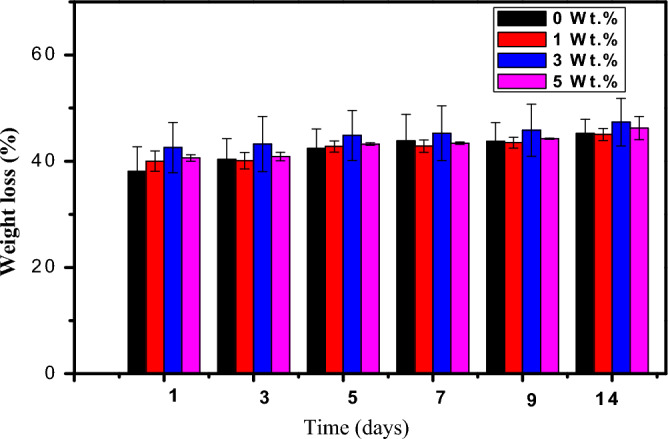
Mean values of percentages of weight loss recorded for PVA: SA loaded with (0, 1, 3, and 5 wt.% TiO_2_-NPs) versus the incubation time.

Figure 7 illustrates that the weight loss percentage of the PVA:SA:TiO_2_ nanocomposite films increases compared to the PVA:SA blend film. The sample containing 3 wt.% of TiO_2_ NPs showed the most tremendous change in weight loss percentage, which dropped as the concentration of TiO_2_ nanoparticles increased, but remained higher than that of the PVA:SA blend. The stability of the PVA:SA:TiO_2_ nanocomposites decreases due to the observed increase in the percentage degradation rates of the prepared nanocomposite films (Fig. 3 and Table 1). The weight loss in the samples increased gradually with increasing the immersion time in PBS. Results also showed that higher content of TiO_2_ NPs in the film (5 wt.%) caused a decrease in weight loss. This can be ascribed to the additional interactions between TiO_2_ NPs, PVA, and SA. According to the swelling and degradation studies, the polymeric matrix's stability is increased by adding TiO_2_ NPs, which may act as a crosslinking agent.

### Antimicrobial activity

Since bacteria are the primary factor influencing wound healing, wound dressings need to have antibacterial properties. Wound dressings with antibacterial properties can inhibit microbial growth and avoid infection^75^. Using the agar diffusion method, Streptomycin antibacterial discs showed inhibition zone of approximately 1.8 cm against all three bacterial strains under study, while Fluconazole anti-fungal discs have inhibition zone of about 1.7 cm against both *Aspergillus Niger,* and *Candida albicans* (yeast). The obtained results show that the antibacterial activity of the prepared PVA:SA:TiO_2_ nanocomposite films was inappropriate to assess by the agar diffusion method. This negative result may be due to the lack of ability of PVA:SA:TiO_2_ nanocomposite to permeate through the agar^76^. Similar results were also reported by Ningrum et al.^77^. Therefore, the broth media method was used to investigate the antibacterial characteristics of the PVA:SA:TiO_2_ nanocomposite films.

**Figure 8:**
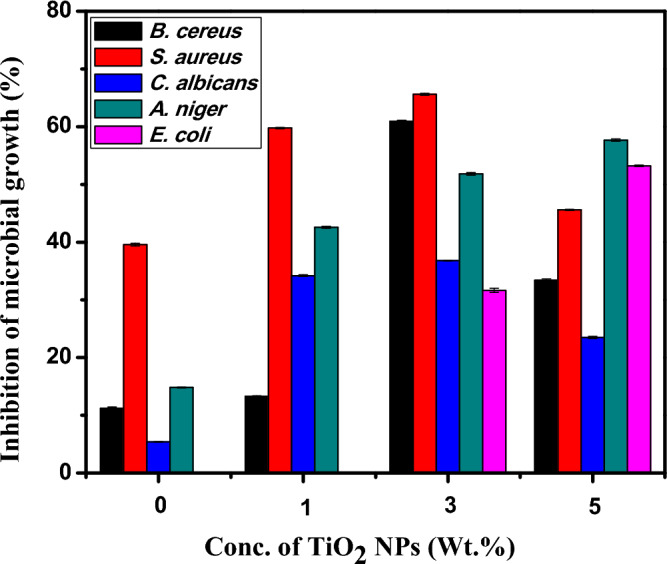
Anti-microbial activity of PVA:SA:TiO_2_ nanocomposite films loaded with different concentrations of TiO_2_ NPs (0, 1, 3, and 5 wt.%) against various microorganisms.

All the tested samples must be inserted in a liquid medium in the broth medium method. Consequently, because this approach allowed the active component to spread through the culture medium, it was more appropriate for the antimicrobial assay^76^. Figure 8 and Table 3 demonstrate the antimicrobial characteristics of the prepared nanocomposite films investigated on various bacterial strains.

**Table 3:** The antimicrobial effect of the prepared nanocomposite films on different bacterial strains.

The concentration of TiO_2_ NPs doped in PVA:SA(wt.%)	Inhibition of microbial growth (%)				
	Microbes				
	*Bacillus cereus*	*Staphylococcus aureus*	*Candida albicans*	*Aspergillus* *niger*	*E. coli*
0	11.23 ± 0.21	39.58 ± 0.25	5.40 ± 0.07	14.84 ± 0.07	-
1	13.30 ± 0.09	59.78 ± 0.11	34.21 ± 0.17	42.58 ± 0.18	-
3	60.93 ± 0.15	65.59 ± 0.18	36.82 ± 0.05	51.82 ± 0.24	31.66 ± 0.35
5	33.41 ± 0.22	45.59 ± 0.08	23.50 ± 0.16	57.66 ± 0.19	53.25 ± 0.13
Positive control	30 ± 0.00	30 ± 0.00	28 ± 0.00	28 ± 0.00	30 ± 0.00

For PVA:SA blend film, *S. aureus* showed the most significant growth inhibition (about 39%), meanwhile, no antibacterial activity against *E. coli* after 24 h of incubation was observed. This indicates that *E. coli* is more resistant to the PVA:SA blend film. The antimicrobial activities of PVA:SA:TiO_2_ nanocomposite films improved compared to the pure PVA:SA blend film. There is a highly significant difference (p < 0.0001) in the antimicrobial effect between the prepared PVA:SA:TiO_2_ nanocomposite films and the positive control group. Increasing the concentration of TiO_2_ NPs to 3 wt.% was found to enhance the antibacterial activity against *B. cereus*, *S. aureus* bacteria, and *A. niger*. This may be ascribed to the gradual release of TiO_2_ NPs from the composite films over time, which is consistent with the degradation results of the nanocomposites.

Additionally, it was observed that the nanocomposite films composed of PVA:SA:TiO_2_ exhibited a superior antibacterial efficacy against *B. cereus* and *S. aureus*, both of which are classified as Gram-positive bacteria, compared to their effectiveness against *E. coli*, a Gram-negative bacterium. The manner of cell membrane penetration may be responsible for the variation in the inhibitory effect of the nanocomposite films against Gram-negative and Gram-positive bacteria. The phospholipids and lipopolysaccharides that constitute the inner and outer leaflets of Gram-negative bacteria's outer cell membrane are less sensitive to antimicrobial agents and can serve as a barrier to permeability, decreasing cell absorption. Gram-positive bacteria have complex cell wall structures and lack a lipopolysaccharide layer, which causes a decrease in their effective action^78^.

A previous study indicates a potential for negatively charged microorganisms to be drawn towards positively charged surfaces of TiO_2_ NPs through electromagnetic forces. This attraction has the potential to result in the oxidation and subsequent destruction of the bacteria. Nanomaterials could destroy DNA and the cellular enzymes by interacting with electron-donating groups. This results in pits in the cell walls of bacteria, which increase permeability and cause cell death^79^. The results illustrate that PVA:SA:TiO_2_ nanocomposite films have a broad-spectrum and efficient antibacterial activity that benefits the wound healing process.

### In vitro* cytotoxicity*

**Figure 9:**
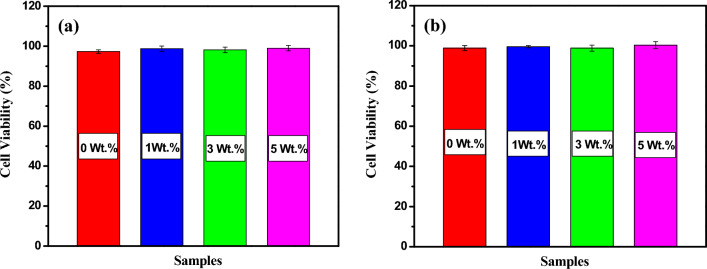
(**a**) In vitro cytotoxicity of 100 µg/ml of the prepared samples applied on HSF (human skin fibroblast), (**b**) in vitro cytotoxicity of applying 50 µg/ml of each sample on the HSF after 24 h.

Biocompatibility is a crucial factor for the material's medical applications, and hence, samples' cytotoxicity needs to be considered. In vitro, the variation of cytotoxicity of the prepared PVA:SA:TiO_2_ nanocomposite films with the human skin fibroblast (HSF), using the SRB assay and two various concentrations of each sample (100 and 50 µg/ml), are displayed in Fig. 9a,b respectively. Blank control group was set as 100% viability. Applying 50 µg/ml of each sample (0, 1, 3, and 5 wt.% of TiO_2_ NPs) on cells did not cause a toxic effect, the estimated cell viability in the presence of those samples was 98.9, 99.58, 98.85, and 100.32%, respectively as revealed in Fig. 9b. It can be observed that PVA:SA:TiO_2_ nanocomposite films showed slightly higher cell viability than the host blend of PVA: SA.

**Figure 10:**
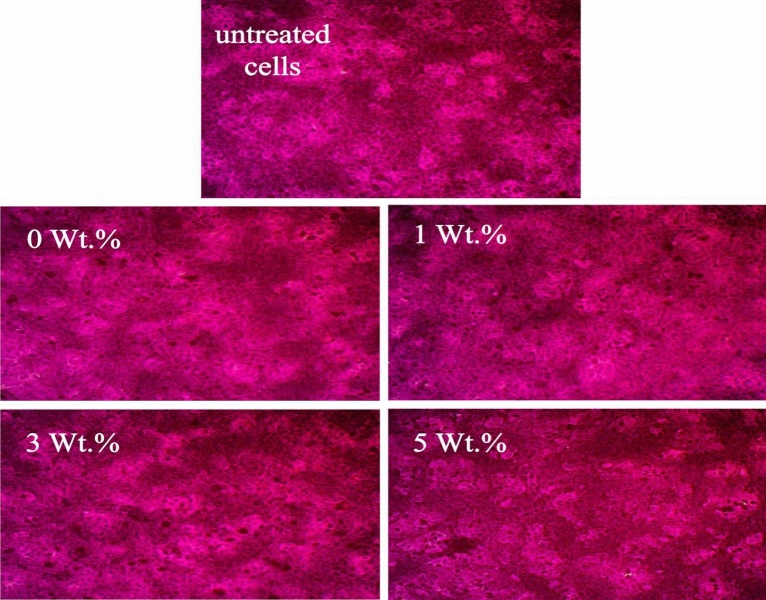
Cell morphology after applying 100 µg/ml of the prepared samples relative to untreated cells.

Increasing the applied dose of PVA:SA:TiO_2_ nanocomposite films containing various contents of TiO_2_ NPs (0, 1, 3, and 5 wt.% ) up to 100 µg/ml (Fig. 9a) caused a non-significant reduction in cell viability up to 97.31, 98.74, 98.18 and 99%, respectively. Although, at higher doses of samples, cell viability decreased, and they were not toxic to cells. These results suggest that adding greenly synthesized TiO_2_ NPs using *Aloe vera* leaf extract into the PVA:SA matrix improved cell response compared to the pure PVA:SA blend. The cell attachment is better than the hydrophilic and positively charged substrate because it can hold cell adhesion-promoting TiO_2_ particles. Certain areas on these molecules are available for cell adhesion^80^. Figure 10 illustrates the typical cellular structure observed after exposing the cells to a substantial concentration (100 µg/ml) of PVA:SA:TiO_2_ nanocomposite films. The present study investigated the effects of the different concentrations of TiO_2_ NPs on nanocomposite films. The concentrations of TiO_2_ NPs used were 0, 1, 3, and 5 weight percent (wt.%) with untreated cells. Moreover, as seen in Fig. 10, following a 24-h incubation period, there are more cells on all samples. This suggests that the film's surface is better for cell adhesion, growth, and proliferation.

Several prior studies have documented that TiO_2_ NPs manufactured using environmentally friendly methods exhibited negligible cytotoxicity when tested on various cell types. The survey conducted by Al-Shabib et al.^81^ showed that the TiO_2_ NPs manufactured using environmentally friendly methods exhibited cytotoxicity towards human hepatocellular carcinoma cells in a dose-dependent manner. Still, they were safe to standard human embryonic kidney cell lines up to 100 μg/ml. Abdel Fadeel et al.^47^ reported that, at concentrations from 0.01 to 100 μg/ml, the greenly synthesized TiO_2_ NPs were nontoxic to breast adenocarcinoma cell and normal human skin fibroblast. Based on the positive results of the biocompatibility test, it can be concluded that PVA:SA:TiO_2_ nanocomposite films studied here had tremendous potential for use as wound dressing materials.

## Conclusions

This study investigates the impact of varying the proportion of TiO_2_ nanoparticles on the thermal, mechanical, biocompatibility, and antibacterial characteristics of PVA:SA nanocomposite films. The Fourier Transform Infrared (FTIR) analysis provided evidence that the Titanium Dioxide Nanoparticles (TiO_2_ NPs) were successfully integrated into the Polyvinyl Alcohol: Sodium Alginate (PVA:SA) matrix. The incorporation of TiO_2_ NPs into the polyvinyl alcohol/sodium alginate (PVA/SA) blend resulted in a decrease in its enthalpy. Furthermore, the glass transition temperatures of the nanocomposites, which consist of varying concentrations of TiO_2_ NPs, were observed to be lower than the glass transition temperature of the matrix material. This observation is supported by the differential scanning calorimetry (DSC) analysis results. The incorporation of TiO_2_ NPs decreased the thermal stability of the polymer blend consisting of PVA and SA. The nanocomposite, doped with a weight percentage of 5% of TiO_2_ NPs, exhibited a greater loss modulus than the remaining samples. Based on the DMA measurements, it was observed that the neat blend of PVA and SA showed the most significant loss factor value within the glass transition zone. Nevertheless, when the doping of TiO_2_ increased, there was a noticeable drop in the height of the loss curve. Due to the high negative zeta potential of TiO_2_ NPs, they demonstrate exceptional stability when dispersed in aqueous solutions, hence preventing any form of aggregation. The addition of TiO_2_ NPs to the polymeric matrix in the swelling and degradation investigation has been found to enhance the stability of the matrix, potentially acting as a crosslinking agent. PVA-SA-TiO_2_ nanocomposite films exhibit various antibacterial properties, effectively targeting multiple bacterial strains. These films have been found to significantly contribute to wound healing by promoting an efficient antibacterial response.

### Future insights

The prepared PVA:SA:TiO_2_ nanocomposite films have a significant antimicrobial activity and biocompatibility properties. Further in vivo investigations on these materials using animal model will be carried out in the future studies to understand their real efficiency as wound dressing materials.

## Data availability

The datasets used and/or analysed during the current study available from the corresponding author on reasonable request. All data generated or analysed during this study are included in this published article [and its supplementary information files].
